# How Mixed Relay Teams in Swimming Should Be Organized for International Championship Success

**DOI:** 10.3389/fpsyg.2021.573285

**Published:** 2021-02-24

**Authors:** Santiago Veiga, Jesús Santos del Cerro, Luis Rodriguez, Alfonso Trinidad, José María González-Ravé

**Affiliations:** ^1^Health and Human Performance Department, Universidad Politécnica de Madrid, Madrid, Spain; ^2^Royal Spanish Swimming Federation, Madrid, Spain; ^3^Department of Statistics, University of Castilla La Mancha, Toledo, Spain; ^4^Catalonian Swimming Federation, Barcelona, Spain; ^5^Faculty of Education and Humanities, Universidad Francisco de Vitoria, Madrid, Spain; ^6^Sports Training Laboratory, Faculty of Sports Sciences, University of Castilla La Mancha, Toledo, Spain

**Keywords:** performance, tactics, competition, team, order

## Abstract

The primary goal of the present research was to determine the order of swimmers on a mixed relay team that would ensure the best performance in the Fédération Internationale de Natation (FINA) World Championships held in Kazan (Russia, 2015), Budapest (Hungary, 2017), and Gwangju (South Korea, 2019). The data were obtained from database websites for the 4 × 100 m freestyle and 4 × 100 m medley official results,^1^ including 660 records from 188 entries of finals and 472 preliminary events. The results showed that the fastest swimmers (according to their best season times) were located primarily in the first or second positions of the freestyle relay. The most successful gender strategy for the 4 × 100 m freestyle (57 out of 82 observations) and for the 4 × 100 m medley (29 out of 83) relays was the order male-male-female-female, although no statistical differences were found (*p* = 0.79) for the medley relays. In the 4 × 100 m freestyle, the second (*p* = 0.002; *β* = 1.62) and third (*p* =0.003; *β* = 1.41) relay legs had a statistical effect on the total relay time, whereas in the 4 × 100 m medley, all four relay legs had a statistical effect (*p* < 0.001) on the final performance, the weight of the four strokes being different in heats with respect to the final round. Also, a later position of the first female swimmer or the consecutive position of two female swimmers in the team order significantly affected the relay performance in specific events. Mixed relay events appeared to present specific strategies in comparison to traditional male- or female-only relay lineups.

## Introduction

The international governing body of swimming (Fédération Internationale de Natation, FINA) included two new relay events in 2015 as part of the competition program in the World Championships: the 4 × 100 m freestyle and 4 × 100 m medley mixed events. In these events, of mixed category, each participating team is composed of two men and two women arranged in a free order. One of these new events, the 4 × 100 m mixed medley relay, will also be included in the competition program of Olympic Games in Tokyo scheduled for 2021. In this way, relay races represent almost a quarter of the total number of events swam in World Championships or Olympic Games.

To date, the research on swimming relays have focused on (1) the composition of the male- or female-only relay teams and (2) differences between individual and relay race performances. The relay team order refers to the order in which swimmers are placed within a relay team (McGibbon et al., 2018). Different strategies are used in international relay events according to the swimming capacity of individuals on the team and to the performance levels of the four swimmers (McGibbon et al., 2018; Fischer et al., 2019). Teams tend to place the fastest two swimmers in the first and fourth relay legs with the slowest two swimmers in the second and third legs, although differences are observed from men to women relays and also from medalist to non-medalist teams (McGibbon et al., 2018). For example, successful teams in the 4 × 200 m freestyle relay from World Swimming Championships tended to save their first ranked swimmers for the fourth relay leg. The order in which the relay team is arranged can also affect the pacing strategies of swimmers in their own relay leg. For example, swimmers placed in the second to fourth relay legs tend more frequently to use a fast start lap and positive pacing compared to lead-off leg swimmers in the 4 × 200 m freestyle finals (McGibbon et al., 2020).

Swimming performances in a relay team, compared to those of individual events, do not seem to represent meaningful time improvements. Skorski et al. (2016), comparing individual and relay performances within the same competition, reported that relay swimmers competing in the first leg did not swim faster than in their individual race performance. Only swimmers in the second to fourth relay positions appeared to benefit from a shorter starting block time, due to different start regulations in relays. However, this could depend on the current race position, and the chances of team success could boost a swimmer’s motivation for the group outcome. Indeed, Hüffmeier et al. (2012) observed improved relay performances when the swimmers were highly instrumental for the group’s performance (i.e., later position in the relay) or the team had a good chance of winning a medal. These strategies, according to the individual swimmer’s capacity but also the expected partial race positioning, could be of practical importance in relay events and represent differences between winning and losing (Ward-Smith and Radford, 2020).

With the inclusion of mixed relay races on swimming competition programs, the tactical options for team strategies become wider as coaches must take into consideration not only individual swimming capacity but also other psychological (Deaner et al., 2015), morphological (Zamparo, 2006), energetic (Barbosa et al., 2010), or biomechanical (Pelayo et al., 1996) features of female vs. male athletes. Female swimmers tend to have less pacing variations (Veiga et al., 2019) or rely more on stroking rate to swim fast (Pelayo et al., 1996), although male world records in swimming exceed female records with a mean gap around 8% (Thibault et al., 2010; Millard-Stafford et al., 2018). This implies that time gaps in the middle of the race could be greater for the mixed relays than for male- or female-only events, depending on the swimmers’ gender of each relay line up. Accordingly, mixed relay teams’ strategies could be influenced not only by the expected individuals’ swimming capacity but also by the expected partial race positioning.

Despite these intricate race tactics, no previous studies available have described or examined how mixed relay teams are arranged and should be arranged for greater success. In addition, the lack of scientific studies investigating relay events has been highlighted (Fischer et al., 2019). Therefore, the main purpose of the present manuscript is to describe the relay team composition of mixed relay events during World Championships and thereby determine their best composition in terms of final performance. It is hypothesized that final relay performances would be influenced by the position of the female swimmers, with faster final times for relay teams composed of male swimmers in the first legs.

## Materials and Methods

To assess the relationship between relay-team composition and gender, previous ranking and stroke, we conducted an observational retrospective study in accordance with the Declaration of Helsinki. Thus, we retrieved historical data from databases websites for official results (www.swimrankings.net). The local Ethical Committee of the University approved this research dated November 30, 2016 and, because the data are based on publicly available resources, no informed consent was necessary. Data were retrieved from the 4 × 100 m freestyle and 4 × 100 m medley relay events during Swimming World Championships organized by FINA, being this event scheduled in Kazan (Russia, 2015), Budapest (Hungary, 2017), and in Gwangju (South Korea, 2019). The database of 660 entries contained records from 188 entries of finals and 472 preliminary events. Each entry contained the following variables: total relay time, round (final or preliminary), relay team order (from first to fourth), gender, 4 × 100 m relay event (medley or freestyle), swimmer’s season best time in 100 m, and the 100 m relay leg time for each swimmer.

The statistical analysis was completed using R software (v.3.6.1 for Windows). Descriptive statistics (mean values and coefficients of dispersion) were calculated for all variables included in the present research. The first analysis was made to identify the relationships between the relay team order (first to fourth relay leg) and the swimmer’s season best time. For this, we calculated the Cramer V coefficient with double-entry tables. Then, to find out which was the most successful strategy in terms of the composition of the relay teams, a one-way ANOVA was performed by gender for both the 4 × 100 m freestyle and medley mixed relays. This was accompanied by the calculation and graphical representation of the confidence intervals of the mean time for each type of team composition.

Subsequently, several general linear models were built for each event. In each model, the aim was to explain an objective variable (relay total time) from a set of independent variables consisting of the time of each relay leg, the position of the first female swimmer (first F), and the consecutive variable. This was a dichotomous variable that took the value = 1 when positions of the two women within the relay were consecutive or 0 when they were not. In relation to the variable first F, it represented the position held by the first female swimmer in the team (values 1, 2, or 3). Both variables were used in an effort to determine how setting the relay team order as two consecutive women [female (F); male (M); female-female-male-male (FFMM), male-female-female-male (MFFM), or male-male-female-female (MMFF)] or as the first female swimmer position could sway the overall relay result. The *R*^2^ Coefficients were calculated as well as global significance test for the estimation of these models. This was meant to determine whether the variables included in the models significantly influence each objective variable. The beta coefficients (*β*) showed the degree of change in the outcome variable for every 1 unit of change in the predictor variable. If the beta coefficient is positive, the interpretation is that for every 1-unit increase in the predictor variable, the outcome variable will increase by the beta coefficient value. If the beta coefficient is negative, the interpretation is that for every 1-unit increase in the predictor variable, the outcome variable will decrease by the beta coefficient value. The explanatory variables were evaluated one by one and were generally considered as significant when the value of *p* was less than 5% in the lineal regression model. Model assumptions of normality and homoscedasticity were tested using the Kolmogorov-Smirnov test and residual graphics, respectively. All the residuals showed a satisfactory pattern.

## Results

**Figure 1:**
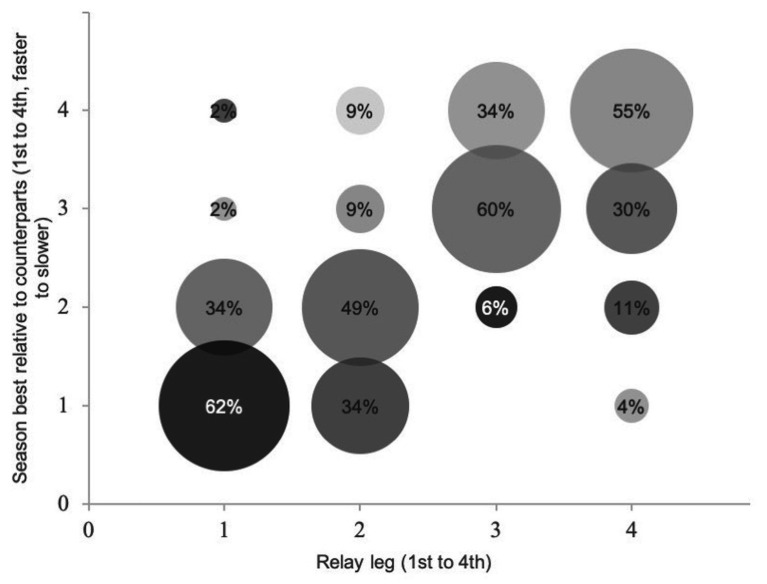
Relay team composition of 4 × 100 m mixed freestyle during 2015, 2017, and 2019 editions of 50 m World Swimming Championships according to the individual’s season best time (1–4, faster to slower) and leg order (first to fourth). M: male and F: female.

The composition of the 4 × 100 m freestyle mixed relay teams during European and World Swimming Championships from 2015 showed a close relationship between the season best time of individual swimmers and their position in the relay leg. As reflected in Figure 1, the fastest swimmers were located primarily (96%) in the first or second relay legs, whereas the slowest swimmers were located predominantly (89%) in the third or fourth relay leg positions. This occurred in all rounds (overall V Cramer: 0.50, heats V Cramer: 0.46) but especially in the finals (V Cramer: 0.59).

**Figure 2:**
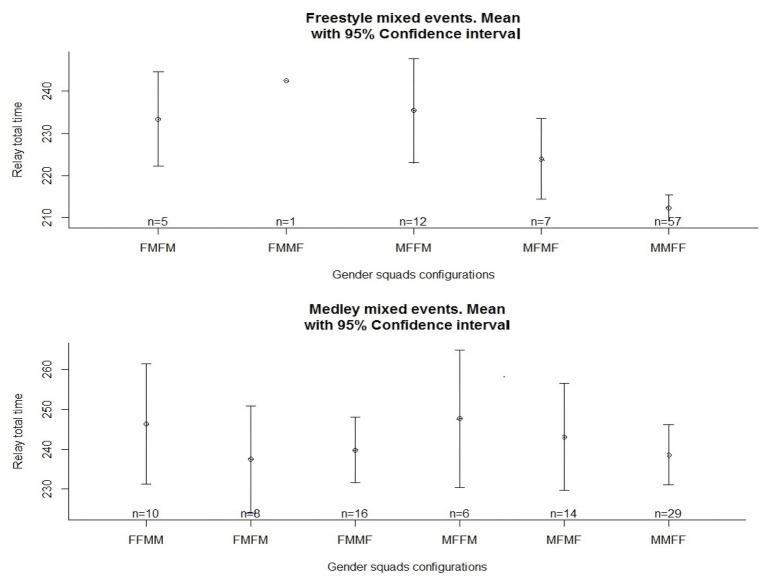
Performance(s) in time (mean ± 95% confidence interval) of different gender squad’s configurations for the 4 × 100 m freestyle (above) and 4 × 100 m medley (below) relay events during 2015, 2017, and 2019 editions of 50 m World Swimming Championships. M: male and F: female.

The most common gender strategy according to one-way ANOVA for both the 4 × 100 m freestyle and medley mixed relays was male-male-female-female order (57 out of 82 cases for the freestyle and 29 out of 83 for the medley relays, respectively). This male-male-female-female relay composition represented better relay total times in the 4 × 100 m freestyle mixed event (*p* < 0.001), whereas no statistical differences (*p* = 0.79) were detected in the 4 × 100 m medley mixed relay (Figure 2). In this latter case, the best relay combination according to final times was female-male-female-male, corresponding to the backstroke-breaststroke-butterfly-freestyle order (237.47 ± 16.14 s). The relative dispersion indicated by the coefficient of variation (CV) of the relay leg performances was not only greater for female than for male swimmers, but also for the heats compared to the final round (CV 6.17 and 1.39% for males vs. 8.38 and 1.46% for females, in the finals or heats round, respectively).

The regression analysis indicated that, in the mixed 4 × 100 m freestyle event (Table 1), the second and third relay leg had a statistical effect on the total relay time, the effect of the second leg being stronger than that of the third (first: *p* < 0.001 and second: *p* < 0.05). Also, there was a statistical effect of the position of the first female swimmers on the team order (*p* < 0.001), indicating a better total relay time when women swam in a delayed order. The problem of the quasi-collinearity did not allow efficient estimations of the regression model parameters for the heats or final round in the 4 × 100 m mixed freestyle.

**Table 1:** Linear regression model for the 4 × 100 m mixed freestyle relay event during 2015, 2017, and 2019 editions of 50 m World Swimming Championships.

		Estimate (*β*)	Std. Error	*t* value	*p*	*R*^2^
Overall	(Intercept)	238.50	4.79	49.47	< 0.001	0.72
	Leg 1	−0.67	0.57	−1.18	0.24	
	Leg 2	1.62	0.51	3.20	0.002	
	Leg 3	1.42	0.47	2.98	0.003	
	Leg 4	0.30	0.48	0.06	0.534	
	First F	−9.39	2.57	−3.65	< 0.001	
	Consecutive	4.45	4.24	1.05	0.297	

First F is the position of the first female swimmers within the relay order and consecutive indicates if position of two female swimmers within the relay were consecutive or not.

For the 4 × 100 m medley event, all four relay legs had a statistical effect on the total relay time (Table 2). However, the freestyle leg showed the greatest influence on the total relay time for the preliminary heats, whereas the breaststroke leg had the greatest influence for the final round. In this event, the first female swimmer position as well as the consecutive position of two female swimmers within the relay team order had a significant impact on the finals but not on the heats.

**Table 2:** Linear regression models for the 4 × 100 m mixed medley relay events during 2015, 2017, and 2019 editions of 50 m World Swimming Championships.

		Estimate (*β*)	Std. Error	*t* value	*p*	*R*^2^
Heats	(Intercept)	247.02	0.46	532.47	< 0.001	0.99
	Leg 1	0.96	0.06	16.28	< 0.001	
	Leg 2	1.04	0.06	16.42	< 0.001	
	Leg 3	0.94	0.06	16.32	< 0.001	
	Leg 4	1.05	0.09	12.14	< 0.001	
	First F	0.36	0.27	1.33	0.190	
	Consecutive	0.75	0.45	1.67	0.101	
Finals	(Intercept)	224.29	0.24	942.24	< 0.001	0.98
	Leg 1	0.86	0.12	7.16	< 0.001	
	Leg 2	1.22	0.09	13.69	< 0.001	
	Leg 3	0.73	0.14	5.07	< 0.001	
	Leg 4	1.07	0.10	10.65	< 0.001	
	First F	−0.74	0.16	−4.65	< 0.001	
	Consecutive	0.96	0.30	3.18	0.005	

First F is the position of the first female swimmers within the relay order and consecutive indicates if position of two female swimmers within the relay were consecutive or not.

## Discussion

The present research was undertaken to determine the most successful order of the team members in mixed relay events according to their best swimming performances and gender. Previous studies have examined the performance and pacing differences between individual and relay swimming events, but no available work has provided insights on how teams should arrange their mixed relay teams, despite that these are new Olympic, World, and European Championships events.

After the compilation of the complete mixed relay events held in 50 m pool World Swimming Championships (since 2015), the main findings of the present study reveal non-significant differences between gender strategy position on the medley relays, but significant differences according to gender in the freestyle relays, with the most suitable combination being male-male-female-female in the teams analyzed. Traditionally, teams tend to locate their fastest swimmers in the first and last relay legs (McGibbon et al., 2018), which, for mixed freestyle relays, would represent male swimmers competing on the first and fourth relay legs. However, our data indicate that the gender variable seems to alter team order strategies (at least in freestyle), as when the two fastest swimmers (males) are located on the first two relay legs, this seems to provide a greater advantage for teams. Indeed, we found a strong relationship between the individual swimming capacity (season best) and the relay leg order, and half of the participating teams arranged their swimmers from the fastest to the slowest (Figure 1). Previous data from 4 × 100 m relays in track running also indicated that the fastest overall times were registered by teams that placed the fastest athlete on the first leg and the two slowest athletes on the last two relay legs (Ward-Smith and Radford, 2020). In the same line, Australia swimming had the fastest time in the world during 2019 with man-man-woman-woman in 4 × 100 mixed freestyle (see footnote 1). This strategy could allow teams to build a large enough lead early in the race to allow the slowest athletes to maintain the leading position (McGibbon et al., 2018). However, little evidence has been found in the literature on relay-team order, and decisions are probably based on the experience of coaches and the characteristics of their swimmers. For example, the United States set the world record of 4 × 100 m mixed freestyle in 2017 with a man-woman-man-woman order. In medley relays, despite no statistical differences, the fastest times were reported by teams that arranged the relay order as female (backstroke), male (breaststroke), female (butterfly), and male (freestyle). This would provide teams a male swimmer on the breaststroke leg, where not only the gender gap (11.31%) seems to be greater according to swimming world records (Thibault et al., 2010) but also to the percentage of time contribution in individual medley events (Saavedra et al., 2012). Of course, in the overall results, the dispersion of relay leg times was greater for the heats compared to the final round. Clearly, teams selected their fastest line up in the finals, whereas they probably reserved some of their fastest swimmers in the heats. A typical improvement from heats to the finals by the same swimmers within a competition could be around 1.2%, (Pyne et al., 2004), which is considerably less than the dispersion of relay times found in the present research. Also, the variation was found to be greater for females compared to males in relay legs, which could indicate that reserves in the round of heats were primarily women or even that the team’s range of performance was greater on the women side.

The regression analysis in the present research indicated that, in mixed freestyle relays, performance in the second and third relay leg (theoretically the slowest) had a statistical effect on the final relay performance. For each additional second in the second or third relay leg, the total relay time decreased 1.62 or 1.42 s, respectively. This is a worthwhile finding given that the fastest swimmers are usually placed in the first relay leg, and it suggests that the range of performance within the relay team should be narrower (McGibbon et al., 2018). In addition, the position of the first female swimmer strongly affected the total relay performance with better relay times for teams who place their female swimmers on the last relay legs. This could be explained by the lower predisposition of women athletes during competition to be more overambitious than males (Deaner et al., 2015), suggesting worse performance of women swimming in a delayed race position (i.e., if placed in the first relay legs against male swimmers) compared to women in the leading race positions (i.e., if placed in the third or fourth relay legs after their male counterparts). Also, previous research has pointed out the importance of the current race position (and the chances of winning a relay race) on the extra effort made by swimmers to swim faster (Hüffmeier et al., 2012). This strategy of placing female swimmers on the last legs of the mixed freestyle relay could provide time gains of practical importance, as observed in previous research with relay-team strategies (Ward-Smith and Radford, 2020).

In the medley relays, all four relay legs (backstroke, breaststroke, butterfly, and freestyle) presented a statistical effect on the total relay performances. However, differences appeared according to the preliminary or final rounds. In the preliminary heats, the freestyle leg presented the greatest influence on the relay time (1.05 s increase in total relay time for each additional second in the freestyle leg) and no influence of the female position on the team order was detected. However, in finals, the breaststroke leg showed the strongest influence on the relay time and the position of the first female also affected total performance. The total race time increased by 1.23 s for each additional second on breaststroke but, in turn, there was a 0.73 s decrease for each delayed position of the first female swimmer. Changes from heats to final performance within a given competition are presumably not influenced by changes in fitness or techniques, so they may be explained by pacing or tactical decisions (Pyne et al., 2004). In preliminary heats, relay teams compete for the final position (not the final time) as they aim to enter the final round (the eight fastest teams regardless of their finishing times), so it is important in the last relay leg (freestyle) to touch the finish wall before the opponents do. This is analogous to the importance of the last race splits on mass start disciplines, where athletes compete for the final position and not time (Rodriguez and Veiga, 2018; Menting et al., 2019). However, in the finals, teams probably compete for the best possible time (Abbiss and Laursen, 2008) in addition to the best possible position, so the slowest leg (breaststroke) is critical for relay performance. In this case, female swimmers who are placed in the last legs of the relay can represent crucial advantages for the final time (Table 2). Previous data on individual medley events (Saavedra et al., 2012) have indicated that men spent a smaller percentage time on the breaststroke than did the women, so this could explain why this relay leg may be crucial for the mixed medley relay performance.

In addition to the relay team order, the consecutive position (or not) of the female swimmer within the relay team order had a statistical effect on the medley relays during final rounds (Table 2). This consecutive placement could have a major impact on the start changeover times, as this is an important factor for relay team’s performance (Skorski et al., 2016). Changeover times comprise the time period from the incoming swimmers touching the wall to the ongoing swimmers leaving the starting block. In mixed relays, the approaching velocity of male swimmers would be considerably faster than females and vice versa (Ribeiro et al., 2019). This could make the outgoing swimmers more difficult to adjust the timing of starting movements when the position of females or males within the relay order would not be consecutive. Indeed, the timing of the starting movements would depend on the gender of the incoming swimmer. This could be especially relevant for mixed relays as changeover times are more important for the final performance in women relays compared to men (Saavedra et al., 2014). However, further research would be needed to confirm this hypothesis.

The present study is limited in terms of the number of events analyzed. This is due to the recent incorporation of these events in different competitions included by the FINA. The upcoming Olympic Games to be held in Japan will increase the data available for the best world-class mixed relays.

## Practical Applications

According to the observed results during European and World Swimming Championships, freestyle and medley mixed relay events appear to present specific strategies in comparison to traditional male- or female-only relay line-ups. Successful teams typically place their fastest swimmers in the first relay legs and, especially on the freestyle relay, male swimmers precede their female counterparts on the relay line-up. This strategy allows female swimmers to perform in a leading race position, which results in an important impact on the total relay performance. Also, the overall success of mixed relay teams appears to rely more on the slowest legs (i.e., the breaststroke leg in medley relays), especially on finals, suggesting that the range of performance within the relay team should be the narrowest. Only on the preliminary heats, where teams try to classify for the finals, the ability of the last team member (freestyle relay leg) to touch the finishing wall ahead of the opponents has the greatest importance on the relay performance.

## Conclusions

During World Swimming Championships, teams participating in the mixed freestyle relay event tend to arrange their line-up from the fastest (first relay leg) to the slowest (fourth relay leg) swimmers. This indicates that the most common gender strategy is a relay sequence of male-male-female-female and this seems to provide statistical advantage in terms of final performance. The second and third freestyle relay legs have a statistical effect on the final performance time, whereas a later positioning of the female swimmers also provides time gains of practical importance. For the mixed medley relay events, there is no advantage in final performance according to typical line-ups, but there is a statistical effect of the position of the first female swimmer, suggesting a later positioning compared to males. Also, performance in the freestyle relay leg during heats as well as on the breaststroke leg during finals has a statistical effect on relay performance. These results provide coaches with critical information, specific to mixed relay strategies, which could represent the difference between winning and losing.

## Data Availability Statement

Publicly available datasets were analyzed in this study. This data can be found at: https://www.swimrankings.net.

## Ethics Statement

The studies involving human participants were reviewed and approved by the Castilla-La Mancha University Ethical Committee. Written informed consent for participation was not required for this study in accordance with the national legislation and the institutional requirements.

## Author Contributions

LR and SV contributed conception and design of the study. AT and JS organized the database. JC, JG-R, and SV performed the statistical analysis. SV and JG-R wrote the first draft of the manuscript. All authors contributed to manuscript revision and read and approved the submitted version.

### Conflict of Interest

The authors declare that the research was conducted in the absence of any commercial or financial relationships that could be construed as a potential conflict of interest.
